# Case report of *enterobacter hormaechei* in sheep with respiratory disease and death

**DOI:** 10.1186/s12917-022-03157-z

**Published:** 2022-01-26

**Authors:** Hongfei Shi, Kun Wang, Li Wang, Shiyu Sun, Bozhen Li, Lunguang Yao

**Affiliations:** 1 Henan Provincial Engineering Laboratory of Insects Bio-Reactor, Henan Provincal Engineering and Technology Center of Health Products for Livestock and Poultry, China-UK-NYNU-RRes Joint Libratory of Insect Biology, Nanyang Normal University, Nanyang, PR China; 2 Henan Provincal Engineering and Technology Center of Health Products for Livestock and Poultry, Key Laboratory of Ecological Security and Collaborative Innovation Centre of Water Security for Water Source Region of Mid-Line of South-To-North Diversion Project of Henan Province, School of Agricultural Engineering, Nanyang Normal University, Nanyang, PR China; 3 Liao Ning Center for Animal Disease Control and Prevention, Shenyang, China

**Keywords:** *Enterobacter hormaechei*, Respiratory disease, Sheep, Drug resistance, Immumohistochemical staining

## Abstract

**Background:**

*Enterobacter hormaechei* is typically a opportunistic pathogenic bacterium in humans, and no pathological change of of *Enterobacter hormaechei* in diseased sheep has previously been documented.

**Case presentation:**

Three free-range, four-month-old female sheep were ill with respiratory disease and died three days after receiving treatment with ceftiofur sodium. A frozen lung sample of one sheep was studied using bacterium isolation, and lung samples of the other two sheep were collected and analyzed by histopathological examination and bacterium isolation. The 16S rRNA gene sequences and biochemical characteristics of the isolates were analyzed. All results showed the isolated strain to be *Enterobacter hormaechei*. Phylogenetic analysis of the 16S rRNA sequence showed three representative strains were most closely related to the strains isolated from calf. Antimicrobial sensitivity tests indicated that no sensitivity to the β-lactam antimicrobials involved in treatment of sheep respiratory disease in China. Detection of the genes responsible for β-lactam resistance showed that all three isolates from sheep harbor bla_SHV_ and bla_KPC._ Interstitial pneumonia, bronchial epithelial cells shedding, and massive mucous secretion were observed in the lung histopathological sections. Immunohistochemical staining showed that specific staining was mainly limited to the alveoli and alveolar septum.

**Conclusions:**

This appears to be the first report of pathological changes in lungs of sheep with respiratory disease and death associated with *Enterobacter hormaechei.*

## Background

*Enterobacter hormaechei* (*E. hormaechei*) is a species of oxidase-negative, gram-negative bacteria widespread in most temperate soils and waters [1]. It is a opportunistic pathogenic bacteria that often causes diseases in immunocompromised hospital patients [2, 3]. In animals, *E. hormaechei* infections have been found in a dead fox with uterine infection [4], piglets with diarrhea [5], calves with respiratory disease [6], pets with respiratory disease complex [7]. Recently in 2021, *E. hormaechei* had been isolated from nasal swabs and ocular swabs collected from goats and sheep [8], however, there is no documentation of pathological change of *E. hormaechei* causing disease in sheep. In this report, *E. hormaechei* strains were isolated from lung samples of three sheep that died of respiratory disease. This is the first report of pathological change of *E. hormaechei* associated with respiratory disease and death in sheep.

Respiratory diseases cause substantial losses in the goat and sheep industry. Many pathogens are involved in respiratory diseases that include caprine pleuropneumonia, contagious bovine pleuropneumonia, Pasteurellosis, mycoplasmosis, streptococcal infections, and hemophilosis [9]. The purpose of the present work was to investigate the cause of the case of three sheep with respiratory disease and present clinical, pathogenic, and molecular findings.

## Case presentation

**Fig. 1:**
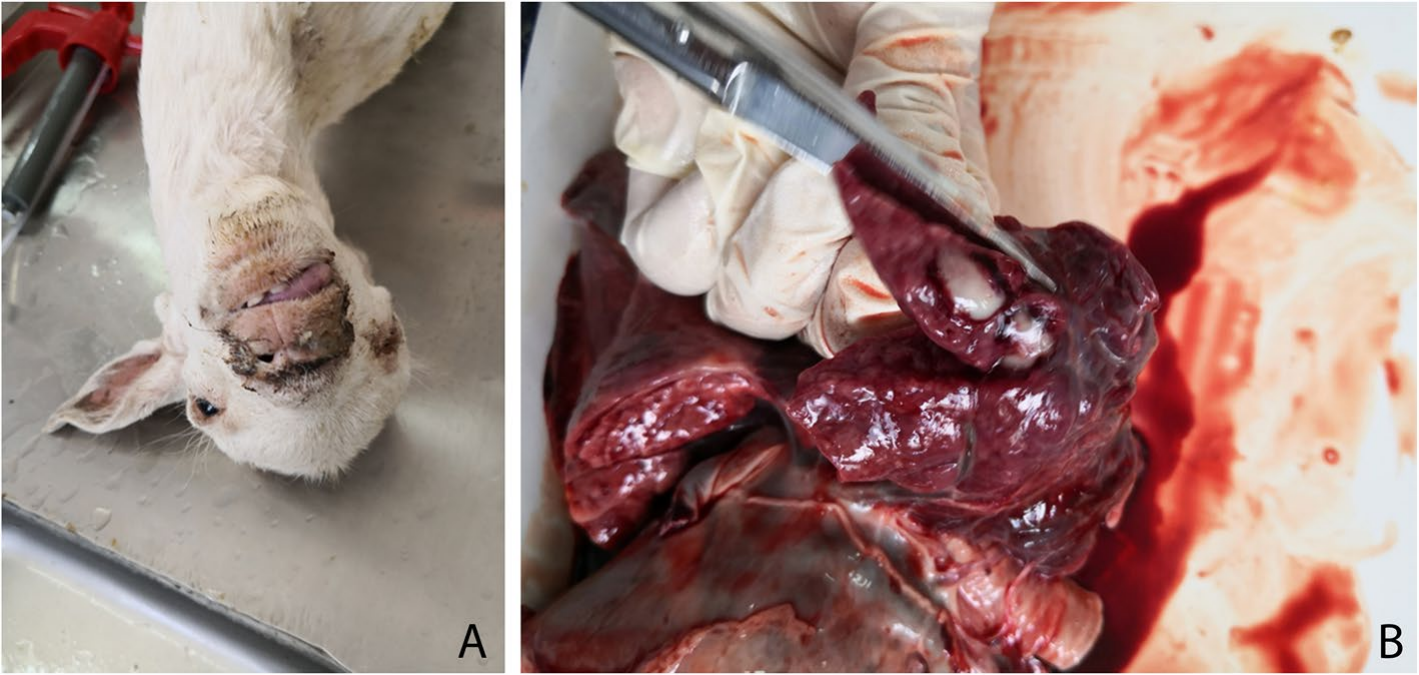
Macroscopic appearance of dead sheep and lungs. **A** Nasal discharge was observed in dead sheep. **B** Lung lesions with pulmonary consolidation and pus suffusion

Three free-range, four-month-old female sheep initially showed nasal discharge and then developed a high fever (41.9–42.3 °C), anorexia, depression, and reduced activity. They were located on a village household farm in Fangcheng City, Henan Province, central China during July 2021. A rural veterinarian found that these sheep had shortness of breath and abnormal vesicular breath sounds, and the results of blood routine index analysis showed an increase of neutrophil count with reference ranges (1.79–2.84 × 10^9^/L). Based on the clinical signs and blood routine index analysis, these sheep were diagnosed as having a respiratory disease caused by a bacterial infection. As lack of the necessary equipment drug susceptibility test had not been done. According to recent veterinary manual in China β-lactam antimicrobials were advised for use in treatment of sheep respiratory disease [10], and therefore under the advice of veterinarian advised, the sick sheep were injected with ceftiofur sodium (Zhusheyong Toubaosaifuna, Hefei Dragon God Animal Pharmaceutical Company) at a dose of 1 mg per kg of b.w. in a single dose given daily for three days. However, one sheep (sheep 1) did not recover and died three days later in the morning. At this time, the other two sheep were near death. Immediately Sheep 1 was dissected by the veterinarian, and its lungs were the only organ with lesions,then the partial lung lesions collected from sheep 1 were frozen and sent to our laboratory with the other dying sheep to determine the cause of death in the same day. At the end of the journey (100 km), the other two sheep had died, and purulent exudate was observed around the nose area (Fig. 1A). After necropsy, all organs were examined. Lungs of sheep 2 and sheep 3 were the only organs with lesions. The lesions consisted of pulmonary consolidation and pus suffusion (Fig. 1B), and there were no visible pathological changes in other organs. The lungs from sheep 1 were frozen, and this sample was subjected to PCR detection and pathogen isolation. The other two lung samples were examined by histopathology and immumohistochemical staining (IHC). As high immunogenicity of ompA protein, the ompA gene was amplified from the genome of the *E. hormaechei* HN18447 strain isolated from a calf in our previous work [6] using the primers 5’- GGAATTCCAACTACAGACTGAGCACGTT-3’ and 5’-CCCAAGCTTGGGGCAAACAACAATGATGGCCCAA-3’. Then amplicons were cloned into vector pET-32a, further the ompA protein was expressed and purified, and used as antigen to immunize rabbit to acquire the ompA protein-specific polyclonal antibodies. At last, polyclonal antibodies were used as the primary antibody, the IHC protocols used followed previously described methods [11].

**Fig. 2:**
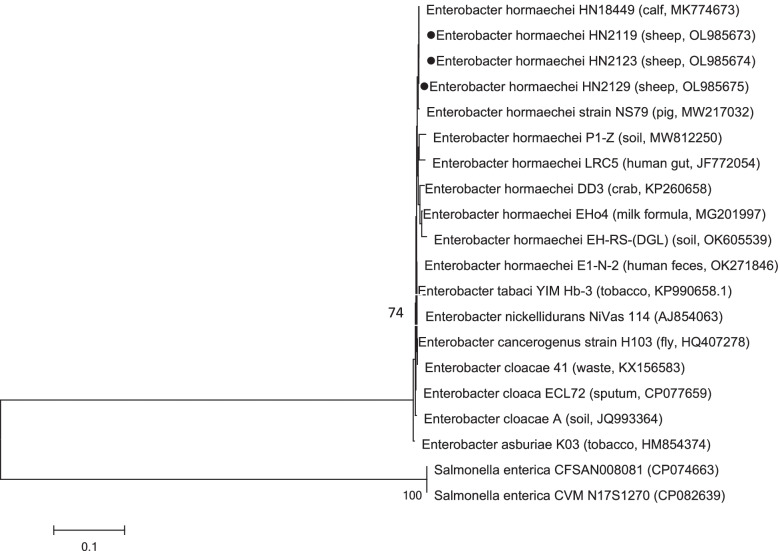
Phylogenetic analysis of *E. hormaechei* from three sheep in China, and reference strains using the 16S rRNA sequences. Phylogenetic analysis was performed using Molecular Evolutionary Genetics Analysis version 6 (MEGA6) based on neighbor-joining criterion and the Kimura 2-parameter model. Stability of the trees was tested by bootstrap analysis using 1,000 replicates. Isolates in this work are highlighted with a symbol (). The hosts (sources) and accession numbers have been indicated in parentheses

Bacteria isolations were done by the streak plate method using three MacConkey agar plates and three nutrient agar plates with 10% calf serum for three sheep lung samples [4, 5]. Round colonies were observed in MacConkey agar plates and nutrient agar plates when samples collected from the three sheep were inoculated and incubated at 37 °C for 24 h using previously described methods [4, 5]. Ten colonies from each plate and total of 60 colonies were chosen for PCR detection and sequencing analysis [4, 5]. General primer sets (5’-AGAGTTTGATCMTGGCTCAG-3’ and TACGGYTACCTTGTTACGACT T) were selected to amplify the 16S rRNA gene in all 60 colonies isolated from three sheep samples [12], and the following standard conditions were used for amplification: initial denaturation at 95 °C for 3 min; 30 cycles of denaturation (30 s at 94 °C), annealing (30 s at 55 °C), and extension (1.5 min at 72 °C); and a final extension at 72 °C for 5 min. The amplified products were recovered from the agarose gel using an EasyPure PCR purification kit (Transgen Biotech, China), and the purified amplicons were directly sequenced in both directions using an ABI automated A373 sequencer (ABI, USA). The results of sequencing the 16S rRNA gene from 60 colonies by two agar plates showed that they shared the same nucleotide sequence. This indicated that the strains isolated from sheep 1, sheep 2, and sheep 3 were the same species. The sequences were compared to existing sequences in the NCBI databases using a BLAST search. The results indicated that the 60 strains were all *E. hormaechei*, and the sequences shared 100% identity to the strains previously isolated from calves [6], and revealed a nucleotide sequence similarity of 98.47%–99.93% to strains from pig (NS79), human (LRC5, E1-N-2), crab (DD3), soil (P1-Z, EH-RS-(DGL)) and milk formula (EHo4). Then three strains (HN2119, HN2123, and HN2129) were selected as representatives of strains isolated from three lungs of sheep 1, sheep 2 and sheep 3 repectively, and sequences of 16S rRNA gene were deposited in GenBank under accession numbers OL985673, OL985674 and OL985675. Phylogenetic analysis of the 16S rRNA sequence (Fig. 2) showed three representative strains were most closely related to the strains isolated from calf (HN18449) and pig (NS79), then secondly related to the strains isolated from soil (P1-Z) and human gut (LRC5), most distantly to the strain isolated from human feces (E1-N-2). To investigate the characteristics of the isolates, three representive strains were further analyzed by biochemical tests and antibiotic sensitivity tests [4, 5]. In addition, to investigate whether other pathogens involve in the infections in the three sheep, TTC-Sabouraud's agar is used for fungus isolation; *caprine parainfluenza virus 3* (CPIV3) [13], *foot and mouth disease virus* (FMDV), *Bluetongue virus* (BTV), *peste des petits ruminants virus* (PPRV), *sheeppox virus* (SPV), *goatpox virus* (GPV), *orf virus* (ORFV) [14], *Mycoplasma ovipneumoniae* [15] were also detected by RT-PCR/PCR. However, no fungus was isolated, and no specific amplicons were obtained by PCR/RT-PCR.

The biochemical tests were performed by using the commercial tubes (Hangzhou microbial reagents, China) according to the instuctions. As showed in Table 1 three isolates were positive for ONPG, ehamnose, typtophan and so on, and negative for indole production, H_2_S, lysine and so on. These characteristics were consistent with those of the *E. hormaechei* as previous report [16].

**Table 1:** Results of the drug susceptibility test

Name	Sensitivity	Diameter			Name	Sensitivity	Diameter		
		HN2119	HN2123	HN2129			HN2119	HN2023	HN2129
Pen	R	0	0	0	Tet	R	0	0	0
Amp	R	0	0	0	Dox	R	0	0	0
Cef	R	0	0	0	Min	R	0	0	0
Amo	R	0	0	0	Fos	S	20	22	19
Cip	S	24	26	27	Nit	R	0	0	0
Enr	R	0	0	0	Rif	R	0	0	0
Nor	R	0	0	0	Azi	R	0	0	0
Lev	R	0	0	0	Met	R	0	0	0
Lo	I/S	20	24	22	Cla	R	0	0	0
Lin	R	6	9	7	Flo	S	29	30	32
Kan	I/S	17	20	18	TriS	S	24	25	26
Gen	I/R	13	8	10	Tri	S	24	24	23
Rox	R	0	0	0	Van	R	0	0	0

*R* Resistant, *I* Intermediate sensitivity, *S* Sensitive, *Diameter* Diameter of inhibitive zone (mm), *Pen* Penicillin G, *Tet* Tetracycline, *Amp* Ampicillin, *Dox* Doxycycline, Ceftiofur sodium, *Min* Minocycline, *Amo* Amoxicillin, *Fos* Fosfomycin, *Cip* Ciprofloxacin, *Nit* Nitrofurantion, *Enr* Enrofloxacin, *Rif* Rifampin, *Nor* Norfloxacin, *Azi* Azithromycin, *Lev* Levofloxacin, *Met* Metronidazole, *Lom* Lomefloxacin, *Cla* Clarithromycin, *Lin* Lincomyci, *Flo* Florfenicol, *Kan* Kanamycin, *TriS* Trimethoprim sulfamethoxazole, *Gen* Gentamicin, *Tri* Trimethoprim, *Rox* Roxithromycin, *Van* Vancomycin

The zone diameter (mm) interpretive criteria for drugs: Penicillin G, Ampicillin and Amoxicillin: S ≥ 17, I:14–16, R ≤ 13, Ciprofloxacin: S ≥ 15, R ≤ 14, Enrofloxacin and Lomefloxacin: S ≥ 22, I:19–21, R ≤ 18, Norfloxacin: S ≥ 17, I:13–16, R ≤ 12, Levofloxacin: S ≥ 17, I:14–16, R ≤ 13, Lincomycin: S ≥ 13, R ≤ 12, Kanamycin: S ≥ 18, I:14–17, R ≤ 13, Gentamicin: S ≥ 15, I:13–14, R ≤ 12, Roxithromycin: S ≥ 13, R ≤ 12, Tetracycline: S ≥ 15, I:12–14, R ≤ 11, Doxycycline: S ≥ 14, I:11–13, R ≤ 10, Minocycline: S ≥ 16, I:13–15, R ≤ 12, Fosfomycin: S ≥ 16, I:13–15, R ≤ 12, Nitrofurantion: S ≥ 17, I:15–16, R ≤ 14, Rifampin: S ≥ 20, I:17–19, R ≤ 16, Azithromycin: S ≥ 21, I:18–20, R ≤ 17, Metronidazole: S ≥ 17, I:14–16, R ≤ 13, Clarithromycin: S ≥ 21, I:18–20, R ≤ 17, Florfenicol: S ≥ 22, I:19–21, R ≤ 18, Trimethoprim sulfamethoxazole: S ≥ 16, I:11–15, R ≤ 10, Trimethoprim: S ≥ 16, I:11–15, R ≤ 10, Vancomycin: S ≥ 16, I:4–8, R ≤ 2

**Fig. 3:**
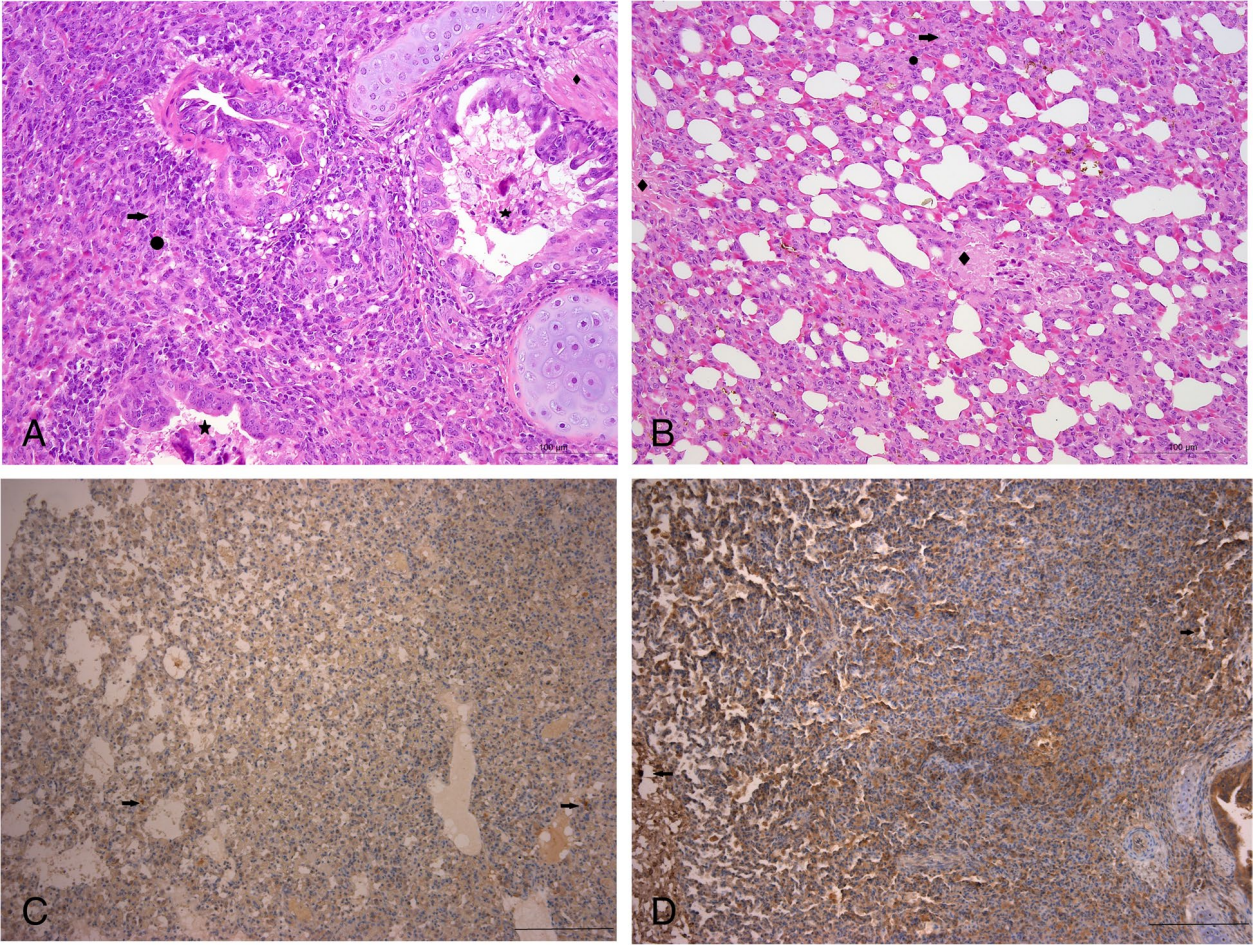
Histopathological section of the lungs and detection of *E. hormaechei* by IHC. **A** Alveoli septum thickening (indicated with arrow), bronchial epithelial cells abscission (indicated with asterisk), mucous secretion (indicated with diamond) and neutrophils infiltration (indicated with circle) of the lungs of sheep 2 (bar = 100 μm, 200 ×). **B** Alveoli septum thickening (indicated with arrow), mucous secretion (indicated with diamond) and neutrophils infiltration (indicated with circle) of the lungs of sheep 3 (bar = 100 μm, 200 ×). **C** Detection of *E. hormaechei* in alveoli and alveolar septum of lungs of sheep 2 by IHC (indicated with arrow) (bar = 200 μm, 100 ×). **D** Detection of *E. hormaechei* in alveoli of lungs of sheep 3 by IHC (indicated with arrow) (bar = 100 μm, 200 ×)

Alveoli septum thickening, bronchial epithelial cells abscission, mucous secretion and neutrophils infiltration in septum thickening region were observed in lungs of sheep 2 as showed in Fig. 3A, and nearly no normal alveoli could be observed in this section, severe interstitial pneumonia of this histopathological feature was in accordance with the macroscopic appearance of dead sheep and lungs. Similarly in lungs of sheep 2 (Fig. 3B) alveoli septum thickening, mucous secretion and neutrophils infiltration were observed. *E. hormaechei* antigen was detected in luminal epithelial cells and alveolar septum in lungs of sheep 2 by IHC (Fig. 3C), in sheep 3 *E. hormaechei* antigen was also detected in luminal epithelial cells (Fig. 3D).

The standards for *E. hormaechei* disk diffusion methods proposed in Clinical and Laboratory Standards Institute (CLSI) guidelines [17] were used to determine growth zone diameter. The results were also interpreted in accordance with CLSI guidelines [17] and the instructions of antibiotic sensitive papers (Hangzhou Microbial Reagent Company, China). A total of 26 antibiotics were tested in the drug susceptibility test. HN2119, HN2123, and HN2129 strains were inoculated on Mueller–Hinton agar plates while *Escherichia coli* strain ATCC 25,922 was used for quality control. The plates were incubated at 37 °C for 18 h in a temperature controlled chamber. The results showed that the three strains were sensitive to ciprofloxacin, fosfomycin, florfenicol, trimethoprim sulfamethoxazole, and trimethoprim, but they were resistant to penicillin G, ampicillin, ceftiofur sodium, amoxicillin, enrofloxacin, norfloxacin, levofloxacin, lincomycin, roxithromycin, tetracycline, doxycycline, minocycline, nitrofurantion, rifampin, azithromycin, metronidazole, clarithromycin, and vancomycin (Table 2). As three *E. hormaechei* strains were resistant to all β-lactam antimicrobials in tests, the presence of the genes responsible for β-lactam resistance were examined by PCR, especially Extended-Spectrum β-Lactamases (ESBL), AmpC, and carbapenemases. In brief, the presence of bla_CTX-M_, bla_SHV_ and bla_TEM_ were detected to confirm ESBL production using primers and amplification conditions as previous work reported [18, 19]. The presence of bla_ACC_, bla_LAT_, bla_CMY_, bla_BIL_, bla_MOX_, bla_DHA_, bla_MIR_, bla_ACT_, and bla_FOX_ were detected to confirm AmpC production using primers and amplification conditions as previous work reported [20]. the presence of bla_IMP_, bla_VIM_, bla_OXA-48_, bla_BIL_, bla_NDM_, bla_KPC_, bla_AIM_, bla_GIM_, and bla_SIM_ were detected to confirm carbapenemases production using primers and amplification conditions as previous work reported [21]. The results showed that all three isolates harbor bla_SHV_ and bla_KPC,,_ and no AmpC-encoding gene was amplified.

**Table 2:** Biochemical reactions of 3 strains of *E. hormaechei*

Test	Resluts			Name	Resluts		
	HN2119	HN2123	HN2129		HN2119	HN2023	HN2129
ONPG ^a^	+	+	+	Rhamnose	+	+	+
Typtophan	+	+	+	Sorbitol	+	+	+
Indole production	-	-	-	Surcrose	+	+	+
Voges-Proskauer	+	+	+	Melibiose	-	-	-
Citrate	+	+	+	Arabinose	+	+	+
H_2_S	-	-	-	Lactose	-	-	-
Urea	+	+	+	Raffinose	-	-	-
Arginine	+	+	+	Dulcitol	+	+	+
Lysine	-	-	-	Esculin	-	-	-
Ornithine	+	+	+	nitrate	+	+	+
Glucose	+	+	+	Oxidase	-	-	-
Mannitol	+	+	+	Motility	+	+	+
3-hydroxybutyrate	-	-	-	3-methyl-D-gluco-pyranose	-	-	-
Putrescine	+	+	+	D-methyl-glycosidase	+	+	+
Inositol	-	-	-	Methyl red	+	+	+

^a^o-Nitrophenyl-,B-D-galactopyranoside

“ + ”: positive

“-”: negative

## Discussion and conclusions

*E. hormaechei* has previously been isolated from blood, bile, pleural fluid, urine, bronchoalveolar lavage fluid and bone marrow samples collected from human patients, and has been found to be associated with respiratory disease, kidney disease, heart disease, meningitis, anemia, and septic shock in patients [1, 3, 22–28]. In domestic animals, infections in foxes, piglets, calves, pets, goats and sheep have been reported [4–8]. However, pathological change of *E. hormaechei* causing disease in sheep or goats was not recorded in previous report. Most studies have shown that *E. hormaechei* is most likely to infect immunocompromised patients [29, 30]*.* We identified *E. hormaechei* associated with respiratory disease and death of three young sheep. Identification was confirmed by 16S rRNA gene sequencing, biochemical tests, and IHC for the clinical samples collected from the lungs of the dead sheep. As no other bacteria was isolated by two plates, no fungus was isolated by TTC-Sabouraud's agar, no virus (CPIV3, FMDV, BTV, PPRV, SPV, GPV and ORFV) and *Mycoplasma ovipneumoniae* was detected by RT-PCR/PCR, the *E. hormaechei* infections should be the cause of death. Histopathological features of the sheep lungs showed mucous secretion in the bronchial lumen. These pathological changes were consistent with the clinical symptoms (nasal discharge) and lung lesions (Fig. 1), Alveoli septum thickening and neutrophils infiltration in sections of lungs of sheep (Fig. 3) were also recorded in lungs of a calf infected by *E. hormaechei*, however, the erythrocyte diapedesis in lungs of calf was not observed in lungs of sheep [6], no data about macroscopic and microscopic changes of lungs from other animals or human could be found, whether these findings in sheep would appear in lungs of other species of animals affected by *E. hormaechei* need further investigations.

*E. hormaechei* was also isolated from lung samples of the three sheep. A case investigation showed that these three sheep had been weaned and grazed in a meadow near a river. Our previous work reported *E. hormaechei* infections in calves within this region [6]. *E. hormaechei* is widespread in most temperate soils and waters, meanwhile in countryside human sewage were used as organic fertilizer for a long time, the *E. hormaechei* from human might spread into the environments where sheep had fed on. These sheep might have been exposed to *E. hormaechei* contaminated grass, soil, or water. Another possibility is that *E. hormaechei* as well as other members of *Enterobacteriaceae* are normal habitants of the respiratory tract of animals [8], and could be an opportunistic pathogenic bacteria combining to some unknown reason, such as summer fever, which cause immunocompromise of the three sheep, at last led to the fatal infections. The Phylogenetic analysis showed strains from sheep in this work were most closely related to the strains isolated from calf in our previous work [6], indicated the potential of same origin; and then secondly related to the strains isolated from soil and human, suggested the origin of these strains isolated from sheep and calves should be human or soil.

Respiratory disease is the most common and costly disease of sheep, and many pathogens, including a variety of bacteria, have been associated with this disease [9]. However, sheep respiratory disease associated with *E. hormaechei* has not been previously reported. Clinical signs observed in this case were similar to the signs in other cases of sheep infected by *Pasteurella* or other bacterial pathogens. Owing to the lack of laboratory diagnosis equipment in rural veterinary clinics in China, misdiagnosis and inappropriate treatment protocols might be made by veterinarians. In a recent veterinary manual the drugs advised for use in treatment of sheep respiratory disease in China include β-lactam antimicrobials [10]. In our report, three sheep were treated with ceftiofur sodium for three days based on the clinical experience of the rural veterinarian. However, the diseased animals still died. After diagnosis by our laboratory, the *E. hormaechei* strains isolated from the lungs of the three sheep were found to not be sensitive to ceftiofur sodium, so the treatment of sheep with this antibiotic was doomed to fail. The *E. hormaechei* strains were also not sensitive to other β-lactam antimicrobials and 14 other antimicrobials. Coincidentally, the strains of *E. hormaechei* isolated from calves in this area in our previous work showed similar resistance to these antimicrobials [6]. It raises questions as to how this resistance has evolved in a soil-dwelling bacterium, presumably not exposed to these antimicrobials, the potential transfer of resistance genes was one of antimicrobial resistance producing ways, the plasmids mediating resistance in human and animal primary pathogens might pass into *E. hormaeche* in this aera. Of course, the origin of E. hormaechei as being from human sewage could be not eliminated, especially broad-spectrum cephalosporin-resistant strains have been isolated from many patients [1, 25]. Detection of the genes responsible for β-lactam resistance showed that all three isolates from sheep harbor bla_SHV_ and bla_KPC,._ bla_SHV_ was also found in *Enterobacteriaceae* from the respiratory tract of sheep and goat with respiratory disease, but not bla_KPC_ [8]. bla_KPC_ was not detected in strains isolated from pets [7], this the first report of bla_KPC_ identification in animals in China, but bla_KPC_ had been detected in strains isolated from human in China [31], this suggested that the possibility route of transmission of resistance plasmid should be from strains in human to strains in animals, and more public health and safety measures should be adopted to reduce this threat. The strains isolated from the three sheep in this case had broad-spectrum resistance to many antibiotics. The genes mediating resistance about other antibiotics in this strain of *E. hormaechei* are not known, and this issue requires additional study.

This is the first report of pathological changes in lungs of sheep with respiratory disease and death being associated with *E. hormaechei*. The diagnosis was made by bacterium isolation, gene sequencing, and IHC. The epidemiological investigations indicated that thesesheep had grazed on the same meadow near the river that might be a reservoir of this pathogen. This information suggests that *E. hormaechei* might be an opportunistic pathogen to sheep. However, there were only three diseased sheep in this case, and broader investigations of *E. hormaechei* associated with disease in sheep are needed to verify this conclusion. Experimental infections of sheep with the bacteria strains isolated in this study would clarify, and possibly confirm, the pathogenicity of *E. hormaechei*.

## Data Availability

All data generated or analyzed during this study are included in this published article. Sequences obtained in the present study are deposited in GenBank under accession numbers OL985673, OL985674 and OL985675.

## Abbreviations

*E. hormaechei*: *Enterobacter hormaechei*
16S rRNA: 16S ribosomal RNA
IHC: Immumohistochemical staining
CPIV3: *caprine parainfluenza virus 3*
FMDV: *foot and mouth disease virus*
BTV: *Bluetongue virus*
PPRV: *peste des petits ruminants virus*
SPV: *sheeppox virus*
GPV: *goatpox virus*
ORFV: *orf virus*
EBSL: Extended-Spectrum β-Lactamases

## References

[1] O’Hara CM, Steigerwalt AG, Hill BC, Farmer JJ 3rd, Fanning GR, Brenner DJ. Enterobacter hormaechei, a new species of the family enterobacteriaceae formerly known as enteric group 75. J Clin Microbiol. 1989;27:2046–9. 10.1128/jcm.27.9.2046-2049.1989 PMC267735 2778068

[2] Davin-Regli A, Bosi C, Charrel R, Ageron E, Papazian L, Grimont PA, Cremieux A, Bollet C (1997). A nosocomial outbreak due to enterobacter cloacae strains with the e. Hormaechei genotype in patients treated with fluoroquinolones. J Clin Microbiol.

[3] Paauw A, Caspers MP, Leverstein-van Hall MA, Schuren FH, Montijn RC, Verhoef J, Fluit AC (2009). Identification of resistance and virulence factors in an epidemic enterobacter hormaechei outbreak strain. Microbiology.

[4] Shan-Shan W, Yun-Jia S, Xing-Yang C, Cheng-Wei W, Shan-Shan GU, Xin Y, Shuang X, Jun-Wei GE, Hong-Yan C (2017). Isolation, identification and phylogenetic analysis of enterobacter hormaechei from foxes. Chinese Veterinary Science.

[5] Lu-Yao LI, Liu MJ, Teng MM, Wang L, Zhang YX, Liu BQ (2017). Study on the biological characteristics of enterobacter hormaechei. Journal of Animal Science & Veterinary Medicine.

[6] Wang Z, Duan L, Liu F, Hu Y, Leng C, Kan Y, Yao L, Shi H (2020). First report of enterobacter hormaechei with respiratory disease in calves. BMC Vet Res.

[7] Khalifa HO, Oreiby AF, Abd El-Hafeez AA, Okanda T, Haque A, Anwar KS, Tanaka M, Miyako K, Tsuji S, Kato Y (2020). First report of multidrug-resistant carbapenemase-producing bacteria coharboring mcr-9 associated with respiratory disease complex in pets: Potential of animal-human transmission. Antimicrob Agents Chemother.

[8] Khalifa HO, Oreiby A, Abd El-Hafeez AA (2021). Abd El Latif A, Okanda T, Kato Y, Matsumoto T: High beta-lactam and quinolone resistance of enterobacteriaceae from the respiratory tract of sheep and goat with respiratory disease. Animals (Basel).

[9] Chakraborty S, Kumar A, Tiwari R, Rahal A, Malik Y, Dhama K, Pal A, Prasad M (2014). Advances in diagnosis of respiratory diseases of small ruminants. Vet Med Int..

[10] Huanchun Chen XW (2013). Veterinary manual.

[11] Dong XM, Zhu YM, Cai H, Lv C, Gao YR, Yu Z, Xue F (2012). Studies on the pathogenesis of a chinese strain of bovine parainfluenza virus type 3 infection in balb/c mice. Vet Microbiol.

[12] Jiang H, Dong H, Zhang G, Yu B, Chapman LR, Fields MW (2006). Microbial diversity in water and sediment of lake chaka, an athalassohaline lake in northwestern china. Appl Environ Microbiol.

[13] Li W, Mao L, Cheng S, Wang Q, Huang J, Deng J, Wang Z, Zhang W, Yang L, Hao F (2014). A novel parainfluenza virus type 3 (piv3) identified from goat herds with respiratory diseases in eastern china. Vet Microbiol.

[14] He YP, Zhang Q, Fu MZ, Xu XG (2017). Development of multiplex pcr for simultaneous detection and differentiation of six DNA and rna viruses from clinical samples of sheep and goats. J Virol Methods.

[15] McAuliffe L, Hatchell FM, Ayling RD, King AIM, Nicholas RAJ (2003). Detection of mycoplasma ovipneumoniae in pasteurella -vaccinated sheep flocks with respiratory disease in england. Veterinary Record.

[16] Mezzatesta ML, Gona F, Stefani S (2012). Enterobacter cloacae complex: Clinical impact and emerging antibiotic resistance. Future Microbiol.

[17] Clinical and laboratory standards institute. Performance standards for antimicrobial susceptibility testing, 29th edition: CLSI M100-S29; 2018, Wayne, USA.

[18] Xu L, Ensor V, Gossain S, Nye K, Hawkey P (2005). Rapid and simple detection of blactx-m genes by multiplex pcr assay. J Med Microbiol.

[19] Dallenne C, Da Costa A, Decre D, Favier C, Arlet G (2010). Development of a set of multiplex pcr assays for the detection of genes encoding important beta-lactamases in enterobacteriaceae. J Antimicrob Chemother.

[20] Perez-Perez FJ, Hanson ND (2002). Detection of plasmid-mediated ampc beta-lactamase genes in clinical isolates by using multiplex pcr. J Clin Microbiol.

[21] Kock R, Daniels-Haardt I, Becker K, Mellmann A, Friedrich AW, Mevius D, Schwarz S, Jurke A (2018). Carbapenem-resistant enterobacteriaceae in wildlife, food-producing, and companion animals: A systematic review. Clin Microbiol Infect.

[22] Carvalho-Assef AP, Pereira PS, Albano RM, Beriao GC, Tavares CP, Chagas TP, Marques EA, Timm LN, Da Silva RC, Falci DR (2014). Detection of ndm-1-, ctx-m-15-, and qnrb4-producing enterobacter hormaechei isolates in brazil. Antimicrob Agents Chemother.

[23] Daurel C, Fiant AL, Bremont S, Courvalin P, Leclercq R (2009). Emergence of an enterobacter hormaechei strain with reduced susceptibility to tigecycline under tigecycline therapy. Antimicrob Agents Chemother.

[24] Giammanco GM, Aleo A, Guida I, Mammina C (2011). Molecular epidemiological survey of citrobacter freundii misidentified as cronobacter spp. (enterobacter sakazakii) and enterobacter hormaechei isolated from powdered infant milk formula. Foodborne Pathog Dis.

[25] Pereira PS, Borghi M, Albano RM, Lopes JC, Silveira MC, Marques EA, Oliveira JC, Asensi MD, Carvalho-Assef AP (2015). Coproduction of ndm-1 and kpc-2 in enterobacter hormaechei from brazil. Microb Drug Resist.

[26] Rafferty B, Dolgilevich S, Kalachikov S, Morozova I, Ju J, Whittier S, Nowygrod R, Kozarov E (2011). Cultivation of enterobacter hormaechei from human atherosclerotic tissue. J Atheroscler Thromb.

[27] Sampaio JL, Ribeiro VB, Campos JC, Rozales FP, Magagnin CM, Falci DR, da Silva RC, Dalarosa MG, Luz DI, Vieira FJ (2014). Detection of oxa-370, an oxa-48-related class d beta-lactamase, in enterobacter hormaechei from brazil. Antimicrob Agents Chemother.

[28] Yang B, Feng Y, McNally A, Zong Z (2018). Occurrence of enterobacter hormaechei carrying blandm-1 and blakpc-2 in china. Diagn Microbiol Infect Dis.

[29] Rottman M, Benzerara Y, Hanau-Bercot B, Bizet C, Philippon A, Arlet G (2002). Chromosomal ampc genes in enterobacter species other than enterobacter cloacae, and ancestral association of the act-1 plasmid-encoded cephalosporinase to enterobacter asburiae. FEMS Microbiol Lett.

[30] Campos LC, Lobianco LF, Seki LM, Santos RM, Asensi MD (2007). Outbreak of enterobacter hormaechei septicaemia in newborns caused by contaminated parenteral nutrition in brazil. J Hosp Infect.

[31] Lin B, Bin LI, Liu X, Xiaohong XU, Cao Y (2016). Study on the resistance mechanisms of carbapenem-resistant enterobacter cloacae. Chin J Infect Chemother.

